# Anomalous Non-Hermitian Open-Boundary Spectrum

**DOI:** 10.3390/e26100845

**Published:** 2024-10-07

**Authors:** Xi-Xi Bao, Gang-Feng Guo, Lei Tan, Wu-Ming Liu

**Affiliations:** 1 School of Sciences and Arts, Suqian University, Suqian 223800, China; 17172@squ.edu.cn; 2 Lanzhou Center for Theoretical Physics, Key Laboratory of Theoretical Physics of Gansu Province, Lanzhou University, Lanzhou 730000, China; 3 Key Laboratory for Magnetism and Magnetic Materials of the Ministry of Education, Lanzhou University, Lanzhou 730000, China; 4 Beijing National Laboratory for Condensed Matter Physics, Institute of Physics, Chinese Academy of Sciences, Beijing 100190, China

**Keywords:** non-Hermitian, continuum bands, skin modes

## Abstract

For a long time, it was presumed that continuum bands could be readily encompassed by open-boundary spectra, irrespective of the system’s modest dimensions. However, our findings reveal a nuanced picture: under open-boundary conditions, the proliferation of complex eigenvalues progresses in a sluggish, oscillating manner as the system expands. Consequently, even in larger systems, the overlap between continuum bands and open-boundary eigenvalues becomes elusive, with the surprising twist that the count of these complex eigenvalues may actually diminish with increasing system size. This counterintuitive trend underscores that the pursuit of an ideal, infinite-sized system scenario does not necessarily align with enlarging the system size. Notably, despite the inherent non-Hermiticity of our system, the eigenstates distribute themselves in a manner reminiscent of Bloch waves. These discoveries hold potential significance for both theoretical explorations and experimental realizations of non-Hermitian systems.

## 1. Introduction

**Figure 1:**
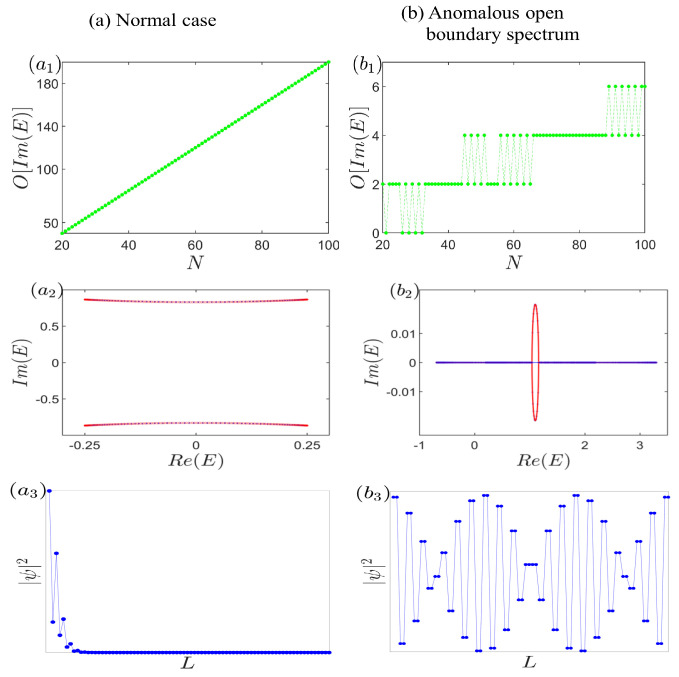
(Color online) Distinguishing features of normal versus anomalous open-boundary spectrum. (**a**) The count of complex open-boundary eigenvalues escalates swiftly and substantially with the expansion of the system size. Remarkably, a seamless integration between the open-boundary spectra (blue curve) and the continuum bands (red curve) can be readily achieved using no more than a few hundred cells. Furthermore, the characteristic non-Hermitian skin effect is prominently displayed. (**b**) In anomalous non-Hermitian open-boundary spectrum, as the system size grows, the number of complex energies under open-boundary conditions either climbs slowly or even diminishes. Notably, even with a system size approaching $O(10^{3})$, the continuum bands struggle to overlap with the open-boundary energies. Regarding the eigenstate distribution, as exemplified in Figure 1($b_{3}$), a unique pattern emerges where $\psi_{n,\alpha }=\psi_{n,\alpha^{\prime }}$ and $\psi_{n,\alpha }=\psi_{2N-n+1,\alpha^{\prime }}$ hold true, with $\left\{\alpha ,\alpha^{\prime }\right\}=\left\{A,B\right\}$. This signifies that the ratio of probability amplitudes between the two sublattices within each unit cell remains constant, underscoring the anomalous nature of this spectrum.

Recently, non-Hermitian topological insulators have garnered significant research attention, with their distinctive characteristics prominently featuring the divergence of complex open-boundary spectra from those arising under periodic boundary conditions and the intriguing non-Hermitian skin effect, where a profusion of eigenstates localize at the boundaries [1,2,3,4,5,6,7,8,9,10,11,12,13,14,15,16,17,18,19,20,21,22,23,24,25,26,27,28,29,30,31,32,33,34,35,36,37,38,39]. To unravel these enigmatic phenomena, innovative concepts such as the generalized Brillouin zone, biorthogonal eigenfunction sets, and continuum bands have emerged [36,37,38,39,40]. In prior investigations, the generalized Brillouin zone has played a pivotal role due to its links to topological invariants and the non-Hermitian skin effect. Regarding the continuum bands, initially postulated in the thermodynamic limit [36,37,38,39,41,42,43,44,45,46,47,48,49,50,51,52,53,54,55,56,57,58,59], it was commonly assumed that they could be readily encompassed by open-boundary spectra, even in relatively small systems (on the order of $O(10^{1})$ sites), as exemplified in Figure 1a. Furthermore, in numerical simulations and experimental designs, it was often presupposed that enlarging the system size would bring us closer to the ideal scenario of an infinite system.

In this work, we delve into the intricate interplay between Hermitian subsystems weakly coupled by a non-Hermitian term, observing a transition in the characteristic polynomial f(z,E) from reducibility to irreducibility. Our findings reveal an intriguing oscillation in the slow growth of open-boundary complex eigenvalues with system size expansion. Notably, as depicted in Figure 1b, even for systems approaching $O(10^{3})$ in size, the count of complex energies under open-boundary conditions barely surpasses 20, rendering them virtually insignificant compared to the imaginary component of continuum bands. Intriguingly, the number of these complex eigenvalues paradoxically diminishes as the system expands, highlighting a counterintuitive phenomenon, i.e., as the system size increases, the results may diverge further from the idealized scenario of infinite size. Remarkably, despite the inherent non-Hermiticity of our system, no skin effect emerges; instead, the eigenstate distribution exhibits a Bloch-wave-like pattern, underscoring the unique physics at play.

This paper is organized as follows. A paradigm and the theoretical framework are constructed in Section 2. Section 3 focuses on the anomalous non-Hermitian open-boundary spectrum. In addition, we demonstrate the distribution of the eigenstates. The conclusion and discussion are found in Section 4.

## 2. Model and Theory

**Figure 2:**
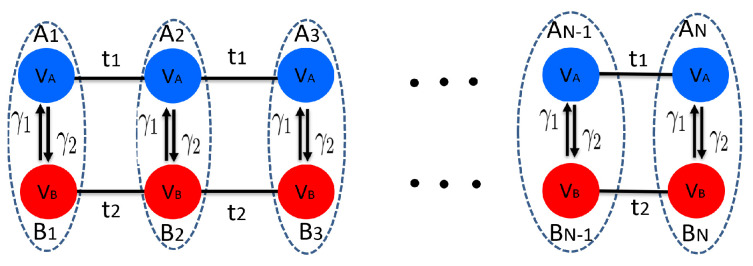
(Color online) Schematic representation of the non-Hermitian coupled chains. The dotted ellipse indicates the unit cell, in which the blue and red circles stand for *A* and *B* sublattice sites, respectively. For *A* (*B*) chain, the hopping amplitude is $t_{1}$ ($t_{2}$) and the onsite potential is $V_{A}$ ($V_{B}$). Two Hermitian chains are coupled by $\gamma_{1}$ and $\gamma_{2}$.

We consider a non-Hermitian system, as shown in Figure 2. Its Hamiltonian reads as

(1) $$\begin{array}{cc} H= & \sum_{n=1}^{N}t_{1}{C^{†}}_{A,n}C_{A,n+1}+t_{1}{C^{†}}_{A,n+1}C_{A,n}+V_{A}{C^{†}}_{A,n}C_{A,n}\\ & +\gamma_{1}{C^{†}}_{A,n}C_{B,n}+\gamma_{2}{C^{†}}_{B,n}C_{A,n}\\ & +t_{2}{C^{†}}_{B,n}C_{B,n+1}+t_{2}{C^{†}}_{B,n+1}C_{B,n}+V_{B}{C^{†}}_{B,n}C_{B,n}, \end{array}$$

where $t_{1}$ and $V_{A}$ represent the hopping amplitudes and onsite potential, respectively, for chain *A*. Similarly, $t_{2}$ and $V_{B}$ correspond to the hopping amplitudes and onsite potential for chain B. Notably,$\gamma_{1}$ and $\gamma_{2}$ are non-reciprocal parameters that couple the two chains, introducing asymmetry in the interactions between them.

Due to space translational symmetry, we can rewrite the Hamiltonian in momentum space as

(2) $$H\left(k\right)=\left(\begin{array}{cc} 2t_{1}\cos\left(k\right)+V_{A} & \gamma_{1}\\ \gamma_{2} & 2t_{2}\cos\left(k\right)+V_{B} \end{array}\right).$$

From this representation, we define the spectral winding number as an integral over the Brillouin zone, given by [38,60,61,62,63,64,65,66]: $W\left(E_{b}\right)=\int_{0}^{2\pi }\frac{dk}{2\pi i}\frac{d}{dk}\ln\det[H\left(k\right)-E_{b}$. The non-Hermitian skin effect is a ubiquitous characteristic of non-Hermitian systems, typically emerging when a non-trivial $E_{b}$ exists for reference energy $E_{b}$. However, for our system, we observe that $W(E_{b})\equiv 0$ for any $E_{b}$ on the complex plane. This finding indicates that despite the inherent non-Hermiticity of our system, the non-Hermitian skin effect is absent, highlighting the unique physics at play.

The characteristic polynomial of our system is

(3) $$\begin{array}{c} f\left(z,E\right)=a_{2}\beta^{2}+a_{1}\beta +a_{0}+a_{-1}\beta^{-1}+a_{-2}\beta^{-2}=0, \end{array}$$

with

(4) $$\begin{array}{cc} & a_{2}=a_{-2}=t_{1}t_{2},\\ & a_{1}=a_{-1}=t_{2}V_{A}+t_{1}V_{B}-E_{OBC}t_{1}-E_{OBC}t_{2},\\ & a_{0}=E_{OBC}^{2}+2t_{1}t_{2}+V_{A}V_{B}-\gamma_{1}\gamma_{2}-E_{OBC}V_{A}-E_{OBC}V_{B}. \end{array}$$

We note that the solutions satisfy

(5) $$\begin{array}{c} \beta_{1}=1/\beta_{4},\beta_{2}=1/\beta_{3} \end{array}$$

because Equation (3) is a reciprocal equation for β. Further, the generalized Brillouin zone is determined by $|\beta_{2}\left|=\right|\beta_{3}|$ [36,37,38,39], which means the generalized Brillouin zone is a unit circle. This result also implies that there does not exist a non-Hermitian skin effect. Further, with the generalized Brillouin zone being determined, the continuum bands $E_{\infty }$ can be obtained by Equation (3).

## 3. Anomalous Non-Hermitian Open-Boundary Spectrum

**Figure 3:**
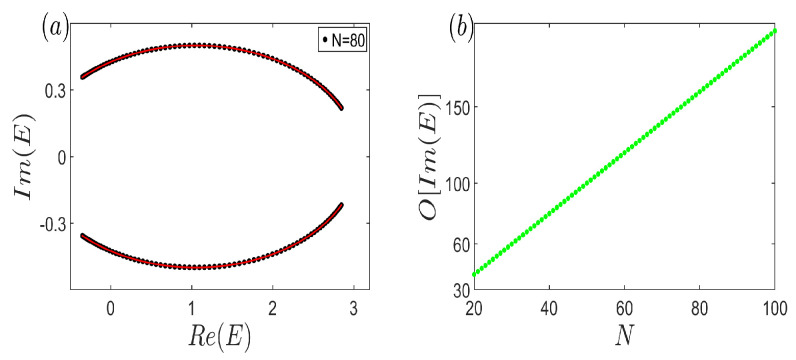
(Color online) (**a**) Open-boundary eigenvalues (black dots) with N=80, and the corresponding continuum bands $E_{\infty }$ (red curve). (**b**) The number of the non-zero imaginary part of eigenvalues under open-boundary condition versus the system size, which grows very rapidly and increasingly. Common parameters are $t_{1}=\frac{1}{2}$, $t_{1}=\frac{1}{2}$, $t_{2}=1$, $V_{A}=\frac{6}{5}$, $V_{B}=\frac{13}{10}$, and $\gamma_{1}=-\gamma_{2}=\frac{3}{5}$.

To elucidate the anomalous non-Hermitian open-boundary spectrum, we commence by showcasing the conventional scenario in Figure 3. Figure 3a vividly demonstrates that the continuum bands $E_{\infty }$ (the red curve) are accurately reproduced by the open eigenvalues (the black dots) for a system size of merely N=80. This underscores the remarkable efficiency of simulating infinite systems using a finite number of unit cells. Furthermore, Figure 3b reveals a striking trend: the count of complex energies under open-boundary conditions escalates rapidly with increasing system size. This observation underscores the fact that larger system sizes yield more precise simulations, aligning with previous research [36,37,38,39,41,50,51,52,53,54,55,56,57,58,67,68,69,70,71,72]. The results reinforce the notion that expanding the system size enhances the fidelity of simulating the infinite system behavior.

**Figure 4:**
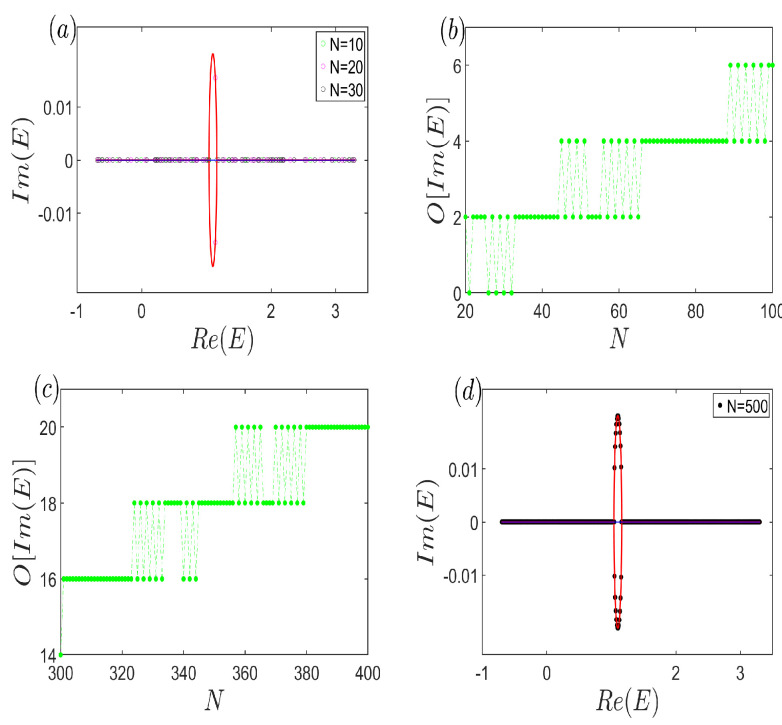
(Color online) (**a**) Continuum bands $E_{\infty }$ with decoupled (blue line) and tiny coupled (red curve) case. The open-boundary energy spectra with different system sizes are also exhibited. (**b**,**c**) The number of the non-zero imaginary part of eigenvalues under open-boundary condition versus the system size, which displays the oscillation behavior. (**d**) Continuum bands (red curve) and open-boundary eigenvalues (black dots). Common parameters are $t_{1}=\frac{1}{2}$, $t_{2}=1$, $V_{A}=\frac{6}{5}$, $V_{B}=\frac{13}{10}$, and $\gamma_{1}=-\gamma_{2}=-\frac{1}{50}$.

We now delve into the anomalous scenario. As depicted in Figure 4a, we contrast the continuum bands $E_{\infty }$ for two distinct cases: $\gamma_{1}=-\gamma_{2}=0$ (the blue line, coinciding with the real axis) and $\gamma_{1}=-\gamma_{2}=-\frac{1}{50}$ (the red curve, spanning the complex plane). Additionally, we present the open-boundary energy spectra at $\gamma_{1}=-\gamma_{2}=-\frac{1}{50}$ for varying system sizes (N=10, 20, and 30). Intriguingly, for a small system size (N=10), the energy spectrum aligns closely with the continuum bands of the decoupled case ($\gamma_{1}=-\gamma_{2}=0$). As the system size increases to N=20, a few open-boundary energies transition to complex values and intersect the continuum bands $E_{\infty }$ of the coupled case. Surprisingly, further enlargement to N=30 reverses this trend, with the eigenenergies retracting to the real axis. This unexpected behavior underscores that increasing the system size paradoxically exacerbates the discrepancy between the open-boundary eigenvalues and the continuum bands.

To visually emphasize the reentrant presence of real energies, Figure 4b plots the count of open-boundary energies with non-zero imaginary parts against the system size. It reveals that for small systems (N<40), the complex eigenvalues oscillate in and out of the complex plane. As the system expands within 40<N<100, the number of complex energies continues to oscillate, albeit with an increasing trend. To underscore the universality of this oscillation, Figure 4c extends the system size range, showcasing a gradual decrease in the number of complex eigenvalues under open-boundary conditions as the system grows larger. Figure 4a–c collectively convey that the increase in complex energies with system size is quite gradual. For a more intuitive grasp of the sparsity of open-boundary spectra on the continuum bands $E_{\infty }$, consider Figure 4d with N=500 (corresponding to L=1000). Even at this significant size, the number of complex eigenvalues barely exceeds 20, indicating their negligibility compared to the continuum bands. Fundamentally, we discover that near the transition point where f(z,E) shifts from reducible to irreducible, the open-boundary spectrum exhibits anomalous behavior, characterized by a slowly growing number of complex energies in an oscillatory manner. This suggests that an increase in the system size does not necessarily lead to a closer approximation of infinite system results, underscoring the intricate interplay between system dimensions and spectral properties.

**Figure 5:**
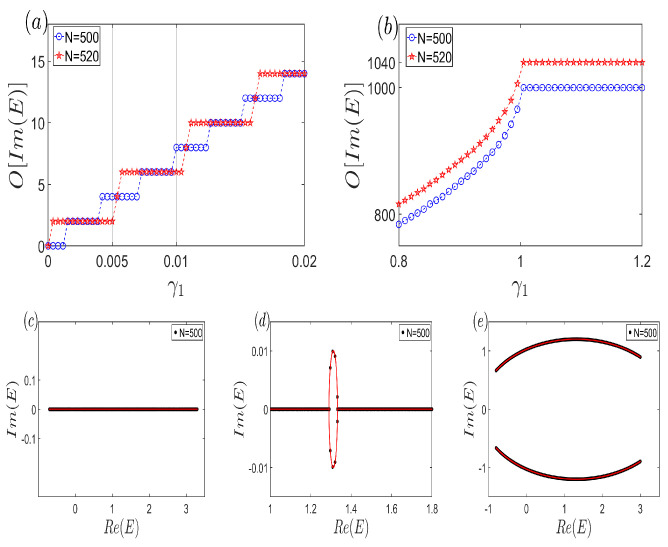
(Color online) (**a**,**b**) The number of the complex open energies under different system sizes versus non-Hermitian parameter $\gamma_{1}$. (**a**) The subsystems are coupled by small couplings, which reflects the oscillatory behavior. (**b**) Subsystems are coupled by large couplings. (**c**–**e**) The overlap between open-boundary spectra and continuum bands. (**c**) $\gamma_{1}=0$. (**d**) $\gamma_{1}=\frac{1}{100}$. (**e**) $\gamma_{1}=\frac{6}{5}$. Common parameters are $t_{1}=\frac{1}{2}$, $t_{2}=\frac{7}{5}$, $V_{A}=\frac{6}{5}$, $V_{B}=1$, and $\gamma_{2}=-\gamma_{1}$.

Another illustrative perspective on the anomalous open-boundary spectrum emerges when examining the effect of varying $\gamma_{1}$. As evident in Figure 5a, for fixed values of $\gamma_{1}$ such as $\frac{1}{200}$ or $\frac{1}{100}$, the count of complex boundary eigenvalues decreases when *N* transitions from 500 to 520, demonstrating the oscillatory pattern in the number of such eigenvalues. Furthermore, Figure 5b highlights a critical threshold in $\gamma_{1}$: beyond a certain value, all eigenvalues disperse across the complex plane. In the decoupled state where $\gamma_{1}=0$ (Figure 5c), the harmonious interplay between open-boundary eigenvalues and continuum bands is evident. However, even a minute deviation from $\gamma_{1}=0$ in Figure 5d results in a negligible number of complex open energies compared to the continuum bands. Further, as $\gamma_{1}$ increases (Figure 5e), the continuum bands become effectively overshadowed by the proliferation of open eigenvalues.

**Figure 6:**
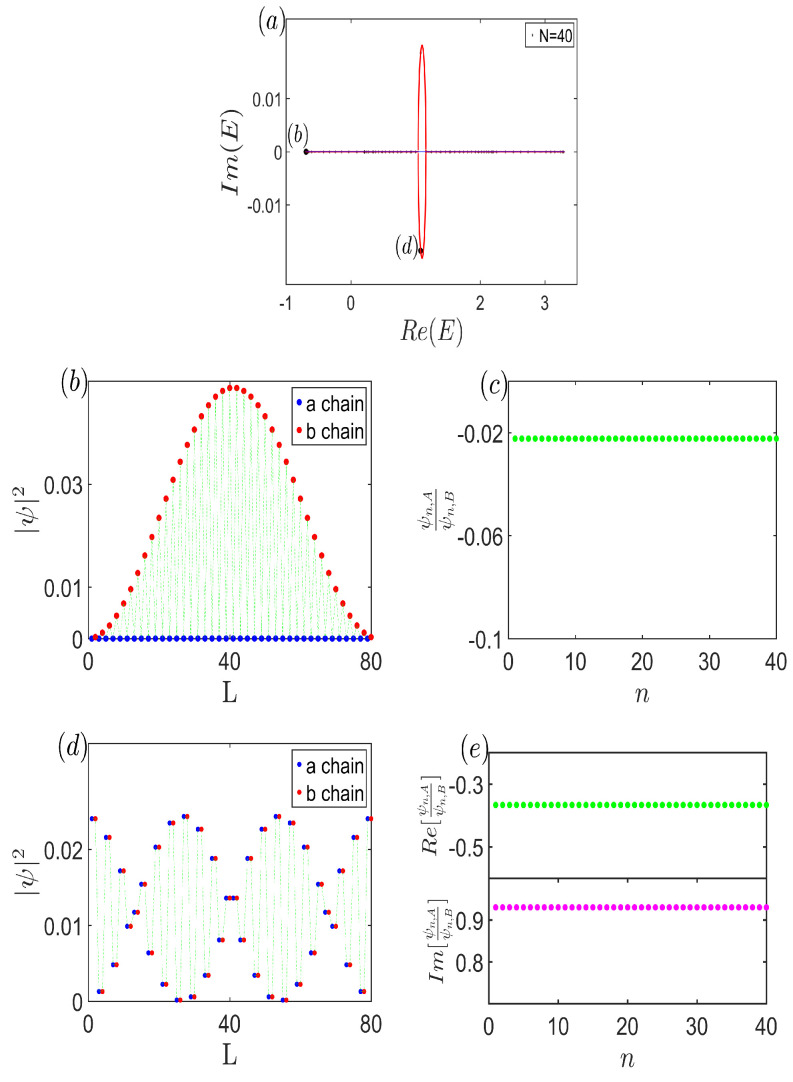
(Color online) (**a**) Continuum bands $E_{\infty }$ with decoupled (blue line) and coupled (red curve) case. The open-boundary energy spectrum with N=40 is also displayed. (**b**) Distribution of the eigenstate corresponding to the real open eigenvalue. (**c**) Ratio of the probability density in every unit cell. Clearly, it is a real constant for the system. (**d**) Distribution of the eigenstate corresponding to the complex open eigenvalue. (**e**) Ratio of the probability density per unit cell, being a complex constant for the system. Common parameters are $t_{1}=\frac{1}{2}$, $t_{2}=1$, $V_{A}=\frac{6}{5}$, $V_{B}=\frac{13}{10}$, and $\gamma_{1}=-\gamma_{2}=-\frac{1}{50}$.

In anomalous circumstances, delving into the distribution of the wave function offers valuable insights. For clarity, Figure 6a depicts the system under investigation with a size of N=40. Notably, Figure 6b reveals that for eigenstates with real open-boundary energies, their density distribution converges toward the system’s center, deviating from the boundaries. Additionally, Figure 6c underscores a fascinating constancy: $\frac{\psi_{n,A}}{\psi_{n,B}}$ remains a real, constant value throughout the system, implying that for real eigenvalues, the probability amplitudes and their ratios across sublattice sites within each unit cell are purely real. Shifting focus to Figure 6d, which illustrates the eigenstate distribution corresponding to complex eigenvalues, we observe an equilibrium in the particle’s probability between the two sublattices within the same unit cell, indicated by $\frac{|\psi_{n,A}|}{|\psi_{n,B}|}=1$. Further analysis in Figure 6e separates the real and imaginary parts of $\frac{\psi_{n,A}}{\psi_{n,B}}$, revealing that this ratio forms a complex constant. This probability distribution mimics a Bloch-wave-like behavior [73], adding to the richness of the observed phenomena. Intriguingly, when the eigenvalue is complex, we discover a unique symmetry: $\psi_{n,\alpha }=\psi_{2N-n+1,\alpha^{\prime }}$ with $\left\{\alpha ,\alpha^{\prime }\right\}=\left\{A,B\right\}$. This feature is distinct in systems where Hermitian subsystems are interconnected via non-Hermitian terms, highlighting the unconventional behavior induced by such couplings.

As shown above, the anomalous non-Hermitian open-boundary spectrum was explored analytically and numerically in terms of the two-band model. The anomalous behavior can be elucidated by other systems as well. We further consider a three-band system, the Hamiltonian of which is

(6) $$\begin{array}{cc} H= & \sum_{n}[(t_{1}{C^{†}}_{A,n}C_{A,n+1}+t_{2}{C^{†}}_{B,n}C_{B,n+1}\\ & +t_{3}{C^{†}}_{C,n}C_{C,n+1}+H.C.)+\gamma_{1}{C^{†}}_{A,n}C_{B,n}\\ & +\gamma_{2}{C^{†}}_{B,n}C_{A,n}+\gamma_{3}{C^{†}}_{B,n}C_{C,n}+\gamma_{4}{C^{†}}_{C,n}C_{B,n}], \end{array}$$

where $t_{1}$, $t_{2}$, and $t_{3}$ are the hopping parameters of the three chains, respectively. $\gamma_{1}$ and $\gamma_{2}$ stand for the non-Hermitian hopping between chain *A* and chain *B*, while $\gamma_{3}$ and $\gamma_{4}$ are the hopping between chain *B* and *C*.

**Figure 7:**
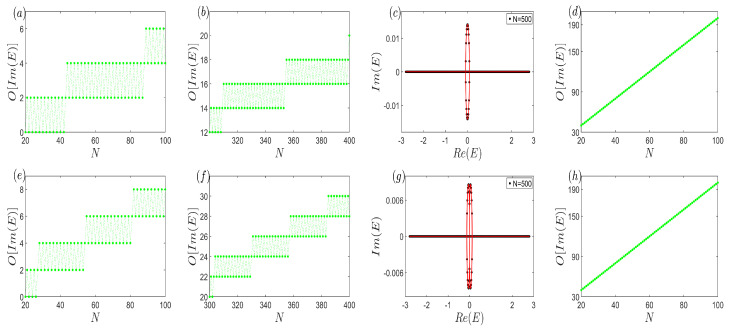
(Color online) Schematic representation of anomalous open-boundary spectrum. (**a**,**b**) The number of the non-zero imaginary part of the open-boundary eigenvalues versus the system size, which displays the oscillation behavior. (**c**) The open-boundary eigenvalues and the corresponding continuum bands. From (**a**–**c**) $\gamma_{1}=-\gamma_{2}=\gamma_{3}=-\gamma_{4}=\frac{1}{100}$. (**d**) The number of the non-zero imaginary part of the open-boundary eigenvalues versus the system size, which increases fleetly and increasingly with $\gamma_{1}=-\gamma_{2}=\gamma_{3}=-\gamma_{4}=1$. (**e**,**f**) The number of the non-zero imaginary part of the open-boundary eigenvalues versus the system size, which exhibits the oscillation behavior as well. (**g**) The open-boundary eigenvalues and the corresponding continuum bands. From (**e**–**g**) $\gamma_{1}=-\gamma_{2}=\gamma_{3}=\gamma_{4}=\frac{1}{100}$. (**h**) The number of the non-zero imaginary part of the open-boundary eigenvalues versus the system size, which also increases fleetly and increasingly with $\gamma_{1}=-\gamma_{2}=\gamma_{3}=\gamma_{4}=1$. The common parameters are $t_{1}=1$, $t_{2}=\frac{6}{5}$, and $t_{3}=\frac{7}{5}$.

In Figure 7a,b, we present the behavior of the imaginary part of the open-boundary eigenvalues as a function of system size when three subsystems are weakly coupled via small non-Hermitian terms ($\gamma_{1}=-\gamma_{2}=\gamma_{3}=-\gamma_{4}=\frac{1}{100}$). Notably, the number of complex eigenvalues exhibits a slow growth pattern accompanied by oscillatory behavior, regardless of the system’s size. This is vividly illustrated in Figure 7c, where the sparse distribution of open-boundary eigenvalues (the black dots) on the continuum bands (the red curve) is shown for a large system size of N=500. For comparison, Figure 7d depicts the scenario where the non-Hermitian coupling strengths are increased to $\gamma_{1}=-\gamma_{2}=\gamma_{3}=-\gamma_{4}=1$. In this case, a marked increase in the number of complex eigenvalues is observed, accompanied by the disappearance of the oscillatory behavior.

Furthermore, as depicted in Figure 7e–h, we consider a scenario where two subsystems (chain *A* and *B*) are coupled via non-Hermitian terms ($\gamma_{1}=-\gamma_{2}$) while the coupling between chain *B* and *C* remains Hermitian ($\gamma_{3}=\gamma_{4}$). For the same system size, the number of complex eigenvalues observed in Figure 7e,f ($\gamma_{1}=-\gamma_{2}=\gamma_{3}=\gamma_{4}=\frac{1}{100}$) is slightly elevated compared to those in Figure 7a,b, but these complex eigenvalues remain virtually inconsequential against the continuum bands. Similarly, Figure 7g showcases a sparse distribution of open-boundary eigenvalues on the continuum bands. Consequently, it remains challenging for the continuum bands to be significantly impacted by the open-boundary energies in this three-band system. Lastly, as evident in Figure 7h, when $\gamma_{1}=-\gamma_{2}=\gamma_{3}=\gamma_{4}=1$, the number of complex eigenvalues increases rapidly, and the oscillatory behavior disappears.

Experimental realization. As we all know, artificial settings [74,75,76,77,78,79,80,81,82,83,84,85,86,87,88], such as cold atoms [74,75,76,77,78,79] and electric circuits [89,90,91,92], possess a high degree of controllability and thus can be engineered to possess dissipation, being the prerequisite to exhibit non-Hermitian behaviors. Therefore, our model can be realized using different types of artificial systems.

## 4. Conclusions and Discussion

In this paper, we delve into an intriguing phenomenon where the number of complex eigenvalues under open-boundary conditions exhibits a slow, oscillating growth pattern as the system size expands. Notably, even when the system size reaches $O(10^{3})$, the count of these complex eigenvalues remains below 20, rendering them virtually negligible compared to the continuum bands. Moreover, we provide analytical insights demonstrating the absence of the non-Hermitian skin effect despite the presence of non-zero non-Hermitian terms. Additionally, we observe that the probability distribution of the open-boundary eigenstates exhibits Bloch-wave-like behavior.

Conventionally, it is often assumed that a modest number of unit cells suffice to capture the essential physical properties of an infinite system in numerical simulations and experimental setups. However, our findings challenge this notion, suggesting that larger system sizes may introduce deviations from the ideal infinite-size scenario. Specifically, the diminishing number of complex eigenvalues with increasing system size indicates the emergence of unforeseen behaviors. Consequently, our results hold significant implications for both theoretical analyses and experimental constructions of non-Hermitian systems.

## Data Availability

The original contributions presented in the study are included in the article, further inquiries can be directed to the corresponding authors.
